# Prevalence of Electrolyte Imbalance in Patients With Acute Stroke: A Systematic Review

**DOI:** 10.7759/cureus.43149

**Published:** 2023-08-08

**Authors:** Md Fahad Hossain, Manish Kharel, Ashma Ul Husna, Mahfuza A Khan, Syed Nurul Aziz, Tamanna Taznin

**Affiliations:** 1 Hospital Medicine, Upazila Health Complex, Ministry of Health, Kishoreganj, BGD; 2 Medicine and Surgery, Jahurul Islam Medical College, Bhagalpur, BGD; 3 Internal Medicine, Mercy Health - St. Elizabeth Boardman Hospital, Youngstown, USA; 4 Internal Medicine, Sylhet MAG (Muhammad Ataul Goni) Osmani Medical College, Sylhet, BGD; 5 Internal Medicine, Shaheed Suhrawardy Medical College, Dhaka, BGD; 6 Medical Education, Chittagong Medical College, Chittagong, BGD

**Keywords:** clinical implications, prevalence, hyponatremia, acute stroke, electrolyte imbalance

## Abstract

Electrolyte abnormalities are common in acute stroke patients and have a substantial impact on the course and prognosis of the disease. Electrolyte imbalances such as hyponatremia, hypokalemia, hypocalcemia, hypomagnesemia, and phosphate abnormalities are frequently seen in this patient population. The incidence, root causes, and medical ramifications of electrolyte abnormalities in acute stroke patients are investigated in this comprehensive study.

According to our research, hyponatremia is the most prevalent electrolyte imbalance. The most common reason for hyponatremia in stroke patients is the syndrome of inappropriate antidiuretic hormone secretion (SIADH). Higher mortality rates, longer hospital admissions, and less favorable functional outcomes are all linked to hyponatremia. Acute stroke patients also typically experience hypokalemia, which affects the severity of the stroke and the recovery of functional abilities. The review furthermore emphasizes the incidence and clinical consequences of hypercalcemia, hypomagnesemia, hypophosphatemia, and hypocalcemia in patients with acute stroke.

The results highlight the significance of early electrolyte imbalance detection and treatment in acute stroke patients. To better comprehend therapeutic approaches, evaluate their influence on stroke outcomes, and analyze prognostic implications, more research is required.

## Introduction and background

The prevalence of electrolyte imbalance in patients with acute stroke has been the subject of extensive research due to its potential impact on stroke outcomes and prognosis. Patients who have experienced an acute stroke frequently have electrolyte abnormalities, which can greatly affect the course of the stroke. In this group, hyponatremia, hypokalemia, hypocalcemia, hypomagnesemia, and phosphate abnormalities are usually seen.

A common electrolyte imbalance seen in individuals with acute stroke is hyponatremia. Hyponatremia was found in 15% of patients within the first 24 hours of stroke start, according to research [1]. Hyponatremia independently predicted 90-day mortality in patients with acute ischemic stroke, according to a different study [2].

Patients with recent strokes frequently have hypokalemia also. Hypokalemia was found in 18.7% of patients with acute ischemic stroke in retrospective research [3]. The study also showed that hypokalemia was linked to lower functional outcomes, greater infarct sizes, and higher stroke severity. Similar findings were made by Barlas et al. (2013) in their study, which discovered that hypokalemia was an isolated indicator of poor functional outcomes three months following a stroke [4].

Acute stroke patients have also been documented to exhibit both hypocalcemia and hypercalcemia. A study indicated that hypocalcemia was present in 11.8% of acute ischemic strokes and was linked to a greater probability of hemorrhagic transformation [5]. Hypercalcemia has also been observed in acute stroke patients. Retrospective research found hypercalcemia in 5.6% of patients with acute ischemic stroke and associated it with more severe strokes and less favorable functional outcomes [6].

Acute stroke-related magnesium abnormalities have also been studied. 10% to 20% of people who have recently suffered a stroke have been found to have hypomagnesemia [7,8]. These investigations demonstrated that hypomagnesemia was linked to worse functional results and bigger infarct sizes. But it's still unclear how exactly hypomagnesemia and stroke severity or death are related. Although less frequent, acute stroke patients with renal impairment or high magnesium delivery have also been found to have hypermagnesemia.

Hypophosphatemia and hyperphosphatemia have received less attention in the context of acute stroke. However, research discovered hypophosphatemia in 5.9% of individuals who had an acute ischemic stroke and discovered that it was linked to a higher three-month death rate [9]. On the other hand, 3.4% of patients with acute ischemic stroke had hyperphosphatemia, which was linked to a greater risk of poor functional outcomes.

Electrolyte imbalances can have serious effects on acute stroke patients since they are essential for sustaining cellular function. It is essential to know the prevalence and cause of electrolyte imbalances for proper therapy and to improve stroke care. Numerous researches have looked at the frequency and characteristics of electrolyte imbalances in this group, giving insight into the possible consequences for therapeutic therapy. In this study, we sought to comprehensively determine the frequency of electrolyte imbalance among patients with acute stroke.

## Review

Method

We followed the PRISMA (Preferred Reporting Items for Systematic Reviews and Meta-Analysis) standards for conducting this systematic review [10].

Data Sources

We have searched various databases which include MEDLINE through Pubmed, Web of Science, The Cochrane Library (Cochrane Central Register of Controlled Trials-CENTRAL), EBSCOhost, and Scopus with a search strategy using the keywords ‘Acute stroke’, ‘brain ischemia’, ‘acute brain Ischemia’, ‘acute brain infraction’, ‘Ischemic stroke’, ‘hemorrhagic stroke’, ‘brain ischemia’, ‘Ischaemic stroke’, ‘acute brain Ischaemia’, ‘Electrolyte imbalance’, ‘Electrolyte disturbances’. The search was done on March 23, 2023.

Study Selection

We searched for articles considering the prevalence of electrolyte imbalance in acute stroke patients. We considered case studies, case series, cross-sectional, case-control, cohort, and clinical trials that are published in the English language only.

We have excluded the study not considering electrolyte imbalance, studies not focusing on acute stroke, laboratory experiments, incomplete or ongoing studies, animal studies, reviews, letters, comments, editorials, book chapters, and opinions.

The title and abstract of the redeemed articles were reviewed individually by two reviewers, and any disagreements were resolved by a third reviewer. To screen the articles, we used the internet tool Rayyan [11]. Then two independent teams went through the full-text articles, and the lead reviewer solved any disagreement. We excluded the articles following the ‘prioritization and sequential exclusion’ technique [12]. Reasons for exclusion were reported.

Data Extraction

We extracted data based on the study type, country, sample size, characteristics, outcome, limitation, and so forth. Two reviewers extracted data independently, and the lead reviewer cross-checked to resolve any dispute.

Data Analysis

We have performed a narrative synthesis here. Due to the variation of findings, a meta-analysis could not be done.

Quality Assessment

The lead author independently assessed the risk of bias in each of the included studies and discussed their assessments with other authors to achieve consensus. The Newcastle-Ottawa scale was adapted for cross-sectional studies and the Newcastle-Ottawa scale cohort version was used to assess the methodological quality of the studies [13-15]. Cross-sectional studies were evaluated using the Newcastle-Ottawa scale modified for cross-sectional studies. The total score was interpreted as follows: 9 to 10 points were deemed very good studies, 7 to 8 points were deemed good studies, 5 to 6 points were deemed satisfactory studies, and 0 to 4 were deemed unsatisfactory studies [13]. The Newcastle-Ottawa scale cohort version was used to assess prospective studies; the interpretation of the total score was: 7 points were considered good studies, 5 to 6 points were considered fair studies, and < 5 points were considered poor studies [14-17].

Results

Search Results

**Figure 1:**
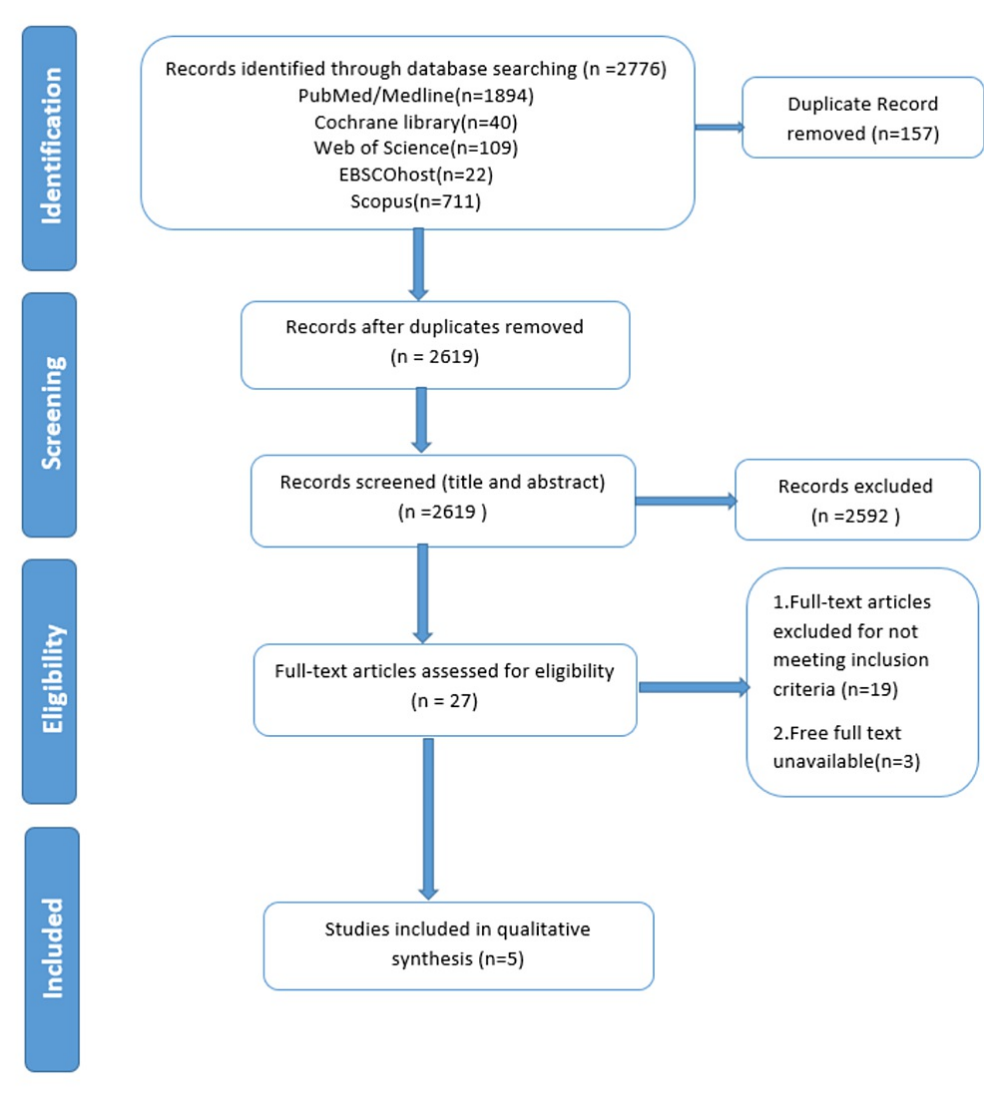
PRISMA flow diagram of the detailed selection process

The comprehensive search from five databases retrieved 2776 articles. After removing 157 duplicates, we listed a total of 2619 articles for screening the title and abstract. At this stage, 2592 articles were excluded due to not meeting inclusion criteria as mentioned in the methods section. Among the remaining articles, 22 were excluded in the phase of full-text screening. Finally, five papers were incorporated into the analysis. PRISMA flow diagram of the detailed selection process of included articles is represented in Figure 1.

Characteristics of Included Studies

Study characteristics for the included studies can be seen in Table 1. Most of the studies followed the cross-sectional design. Most of the studies showed the impairment of sodium in acute stroke. Two studies reported an association between electrolyte prevalence and outcome in stroke, two studies reported common electrolyte abnormality in different kinds of stroke, and one study studied common electrolyte abnormality in ischaemic stroke. One study tried to study the association between electrolyte disorder and prognosis using NIH Stroke Scale. All studies reported that hyponatremia was significantly the most common electrolyte abnormality. One study tried to study the association between electrolyte disorder and prognosis using NIH Stroke Scale.

**Table 1:** Study characteristics for the included studies CSWS: Cerebral salt wasting syndrome; CVA: Cerebrovascular accident; TIA: Transient ischemic attack; NIHSS: National Institute of Health Stroke Scale

Study	Components	Features
Mahesar et al., 2019 [18]	Study design	Cross-sectional study
	Country	Pakistan
	Summary	132 patients diagnosed with hyponatremia were selected to evaluate the degree of hyponatremia and common types of hyponatremia. Mild hyponatremia was a common type of hyponatremia.
	Population	130
	Outcome	Hyponatremia is the most common electrolyte abnormality with prevalence of 39.23%, among which Mild hyponatremia (130-134 mmol/L) is most common (25%). Among the patients developing hyponatremia diabetes mellitus and hypertension is the most common comorbid condition (P<0.001).
	Limitation	Small sample size
Karunanandham et al., 2018 [19]	Study design	Cross-sectional study
	Country	India
	Summary	202 CVA patients were selected among which 163 had ischaemic stroke, 24 had hemorrhagic stroke and 15 had TIA, with medium age 57.5 years and male 86.3%. Hyponatremia is common and SIADH is the most common pathology.
	Population	202
	Outcome	Prevalence of hyponatremia was 38.61% among which 21.28% was ascertained to be SIADH and 7.42% to be CSWS. The mean hospital stay was prolonged in the patient with hyponatremia (21+/-8.51) vs normonatremia (10 +/- 3.8), P=0.04.
	Limitation	Small sample size. Patients enrolled from a single center.
Fofi et al., 2018 [20]	Study design	Prospective cohort
	Country	Italy
	Summary	475 Caucasian patients with stroke (256 males [53.9%], 219 females [46.1%], mean age 67 years ± 15 years; range: 14–96 years) were selected to determine electrolyte abnormality and their effect on mortality and NIHSS score. The alteration of sodium is associated with higher odds of death.
	Population	475
	Outcome	Hyponatremia was reported in 28 (6.3%), and six (20.7%) of the 29 deceased patients, while hyper-natremia was detected in nine (2%) survivors, and two (6.9%) of the deceased patients. Hypokalemia was present in 24 (5.3%) survivors and in one (3.4%) deceased patient, while hyperkalemia was present in 16 (3.6%) survivors and one (3.4%) deceased patient. Multiple logistic regression analysis showed that the baseline NIHSS score (odds ratio [OR] = 1.33; 95% CI = 1.22–1.44) and alterations in serum sodium (OR = 6.89; 95% CI = 1.94–24.40]) were associated with higher odds of death.
	Limitation	Lack of a control group. Short observational period. Not able to determine a relationship between electrolyte imbalance and stroke localization.
Siddiqui et al., 2012 [21]	Study design	Cross-sectional study
	Country	Bangladesh
	Summary	100 patients diagnosed with stroke were selected with a variable age group to determine common electrolytes status, indifferent type of acute stroke patients and their association with some common clinical presentation. Hyponatremia is the most common electrolyte abnormality and hypokalemia is more common in hemorrhagic stroke.
	Population	100
	Outcome	Hyponatremia (32%) is the most common electrolyte abnormality with a statistical significance difference among types of stroke, but hypokalemia (19%) is statistically significant (p<0.05) and more common in hemorrhagic stroke. Headache (74%) is the most common symptom in patients with dyselectrolytemia followed by vomiting (73.46%), vertigo (42.85%), and seizure (2.85%).
	Limitation	The sample size was relatively small. No long-term follow-up could be carried out. Definite causes of this dyselectrolytemia during acute stroke could not carry out.
Mansoor et al., 2021 [22]	Study design	Cross-sectional study
	Country	Pakistan
	Summary	300 patients with stroke were evaluated to determine the frequency of electrolyte imbalance.
	Population	300 patients with stroke among which 139 were from the ischaemic group and 161 were from the hemorrhagic group.
	Outcome	The level of sodium is significantly lower in ischaemic stroke (129.41 +/- 3.12) vs hemorrhagic stroke (134.42 +/- 3.46) P-value <0.01, whereas potassium is significantly higher among hemorrhagic stroke (6.27 +/- 1.12 vs 4.31 +/- 0.71, P-value <0.01)
	Limitation	Single institute. Small sample size. A definite association could not be established.

Quality Assessment Results of the Included Studies

All the cross-sectional studies and one prospective cohort study are considered good studies as cross-sectional studies score 7 and prospective cohort study scores 8 according to the Newcastle-Ottawa scale. Their score breakdowns are listed in Table 2 and Table 3.

**Table 2:** Newcastle-Ottawa scale adapted for cross-sectional studies * Score 1 ** Score 2

First author, year	Selection				Comparability	Outcome		Total
	1	2	3	4		1	2	
Mansoor et al., 2021 [22]	*			**	*	**	*	7
Mahesar et al., 2019 [18]	*			**	*	**	*	7
Karunanandham et al., 2018 [19]	*			**	*	**	*	7
Siddiqui et al., 2012 [21]	*			**	*	**	*	7

**Table 3:** Newcastle-Ottawa scale (prospective cohort study) * Score 1 ** Score 2

First author, year	Selection				Comparability	Outcome			Total
	1	2	3	4		1	2	3	
Fofi et al., 2018 [20]	*	*	*	*	*	*	*	*	8

Discussion

Summary of Findings

The systematic review encompassing multiple articles provides valuable insights into the prevalence, causes, and clinical implications of hyponatremia and electrolyte abnormalities in stroke patients. Mahesar et al. (2019) highlight that hyponatremia is the most common electrolyte abnormality (prevalence of 39.23%) and further note that mild hyponatremia (130-134 mmol/L) is the most common subtype accounting for 25% of cases [18].

Hyponatremia is the most common electrolyte imbalance in stroke and negatively impacts the mortality and functional outcome of stroke. Electrolyte imbalance adversely affects the stroke outcome with negative impacts on mortality and morbidity. To enhance the outcome of a stroke, electrolyte abnormalities must be identified early. Therefore, early electrolyte imbalance identification is crucial for preventing morbidity and mortality in acute stroke patients. The two main causes of hyponatremia are CSWS (cerebral salt wasting syndrome) and SIADH (Syndrome of Inappropriate Antidiuretic Hormone Secretion). Understanding the causes is essential because different management approaches are used. Fluid restriction should be used to treat SIADH, whereas salt and water administration should be used to treat CSW.

Karunanandham et al. (2018) emphasize that the Syndrome of Inappropriate Antidiuretic Hormone Secretion (21.28%) is the most common cause of hyponatremia in patients with stroke [19]. In addition, he also found that the mean hospital stay was prolonged in the patient with hyponatremia (21 +/- 8.51) vs normonatremia (10 +/- 3.8), P=0.04. In addition, Fofi et al. (2018) contribute by reporting a higher prevalence of hyponatremia potential association with poorer outcomes [20]. Moreover, they establish that baseline National Institute of Health Stroke Scale (NIHSS) score and alterations in serum sodium levels are associated with higher odds of death. Hyponatremia was reported in 28 (6.3%), and in six (20.7%) of the 29 deceased patients, while hyper-natremia was detected in nine (2%) survivors, and two (6.9%) of the deceased patients. Hypokalemia was present in 24 (5.3%) survivors and in one (3.4%) deceased patient, while hyperkalemia was present in 16 (3.6%) survivors and one (3.4%) deceased patient. Multiple logistic regression analysis showed that the baseline NIHSS score (odds ratio [OR] = 1.33; 95% CI = 1.22-1.44) and alterations in serum sodium (OR = 6.89; 95% CI = 1.94-24.40) were associated with higher odds of death.

There are two studies that basically focus on electrolyte prevalence in different types of stroke.

Siddiqui et al. (2012) demonstrate that hyponatremia (32%) is the most common electrolyte abnormality with a statistical significance difference among types of stroke, but hypokalemia (19%) is statistically significant (p<0.05) and more common in hemorrhagic stroke [21]. Headache (74%) is the most common symptom in patients with dyselectrolytemia followed by vomiting (73.46%), vertigo (42.85%), and seizure (2.85%).

Lastly, Mansoor et al. (2021) demonstrate that hyponatremia is statistically significantly more common in ischemic stroke compared to hemorrhagic stroke, while potassium levels are significantly higher in hemorrhagic stroke [22]. The level of sodium is significantly lower in ischaemic stroke (129.41 +/- 3.12) vs hemorrhagic stroke (134.42 +/- 3.46), P-value <0.01, whereas potassium is significantly higher among hemorrhagic stroke (6.27 +/- 1.12 vs 4.31 +/- 0.71, P-value <0.01).

This comprehensive discussion highlights the varying prevalence rates and clinical implications of electrolyte abnormalities in stroke patients, providing a more comprehensive understanding of these conditions. In summary, hyponatremia is the most common electrolyte abnormality and has a strong association with outcomes. Moreover, SIADH is one of the most common causes. Early diagnosis and treatment of cerebrovascular accident (CVA) can decrease hospital stay, mortality, and morbidity.

Research Implications

The prevalence and clinical consequences of electrolyte imbalances in stroke patients have several scientific implications that call for additional study.

First, future studies should concentrate on defining the underlying processes and perilous signs of electrolyte abnormalities in stroke patients. Developing specialized preventative and management methods will require an understanding of the pathophysiology and the determination of predisposing variables. Studies examining the key factors contributing to hyponatremia in stroke patients, such as cerebral salt wasting syndrome (CSWS) and Syndrome of Inappropriate Antidiuretic Hormone Secretion (SIADH), might offer important insights into how to manage and treat these disorders [18,19,22,23].

Further research is required to determine how electrolyte imbalances affect outcomes following stroke, such as mortality, functional outcomes, and duration of hospital stay. Prioritizing therapies aimed at enhancing patient care and enhancing prognosis can be made easier by identifying the individual electrolyte abnormalities that have the greatest influence on these outcomes [20,21,24].

Additionally, studies should look at possible therapeutic methods for treating electrolyte abnormalities in stroke patients. Evidence-based recommendations for treating these diseases can be obtained by assessing the efficacy of therapies like fluid restriction for SIADH or salt and water delivery for CSWS [22,25]. It would be beneficial to look into how focused electrolyte correction affects patient outcomes including neurological recovery and quality of life [20,21].

Future research should investigate the underlying processes that cause these discrepancies in light of the variations in electrolyte prevalence between ischemic and hemorrhagic strokes. Understanding the exact causes of increased rates of hyponatremia in ischemic stroke and higher potassium levels in hemorrhagic stroke will aid in the development of specialized therapeutic strategies for the various subtypes of stroke [23,26].

The long-term impact and prognostic implications of electrolyte imbalances in stroke survivors should also be the subject of further study. Thorough knowledge of the long-term effects and the development of preventative measures can be aided by longitudinal studies evaluating the influence of electrolyte imbalances on post-stroke problems, such as recurrent stroke, cognitive decline, and disability [27,28].

In a nutshell, more studies are necessary to better understand the underlying processes, risk factors, therapeutic approaches, and long-term outcomes as a result of the prevalence and clinical consequences of electrolyte imbalances in stroke patients [29,30]. By solving these research gaps, we can better understand these disorders, develop better care plans, and improve the outcomes for stroke patients.

Strengths and Limitations

We adhered closely to the PRISMA recommendations when conducting this systematic review. The Newcastle-Ottawa scale evaluation method was used to critically evaluate the included publications for bias risk.

There are obviously some drawbacks to this study. Only English-language articles were taken into consideration. Therefore, it's possible that studies published in any other language may be overlooked. Second, we only included a small number of articles with adequate sample sizes. Thus, the outcomes might occasionally be inflated.

## Conclusions

Stroke patients frequently have electrolyte abnormalities, especially hyponatremia, which can have important therapeutic ramifications. The systematic review and the research covered in this analysis offer important new information about the prevalence, root causes, and consequences of these electrolyte abnormalities. With moderate hyponatremia being the most often seen subtype, hyponatremia emerges as the most prevalent electrolyte anomaly in stroke. The most common reason for hyponatremia in stroke patients is a syndrome of inappropriate antidiuretic hormone secretion (SIADH). Early detection and treatment of electrolyte imbalances are essential because they can affect patient outcomes such as higher mortality, longer hospital stays, and worse functional results. For the purpose of creating focused therapies and optimizing stroke care techniques, which will eventually improve patient outcomes and lessen the burden of the condition, more study and focus on electrolyte imbalances in stroke patients is required.
